# Molecular diagnosis of *Coxiella burnetii* in culture negative endocarditis and vascular infection in South Korea

**DOI:** 10.1080/07853890.2021.2005821

**Published:** 2021-11-22

**Authors:** Moonsuk Bae, Hyo Joo Lee, Joung Ha Park, Seongman Bae, Jiwon Jung, Min Jae Kim, Sang-Oh Lee, Sang-Ho Choi, Yang Soo Kim, Yong Shin, Sung-Han Kim

**Affiliations:** a Department of Infectious Diseases, Asan Medical Institute of Convergence Science and Technology, Asan Medical Center, University of Ulsan College of Medicine, Seoul, Republic of Korea; b Division of Infectious Diseases, Department of Internal Medicine, Pusan National University Yangsan Hospital, Yangsan, Republic of Korea; c Department of Convergence Medicine, Asan Medical Institute of Convergence Science and Technology, Asan Medical Center, University of Ulsan College of Medicine, Seoul, Republic of Korea; d Department of Biotechnology, Yonsei University, Seoul, Republic of Korea

**Keywords:** Culture negative endocarditis, Q fever endocarditis, polymerase chain reaction, *Coxiella burnetii*

## Abstract

**Background:**

Q fever endocarditis is a major cause of culture-negative endocarditis. The role of *Coxellia burnetii* is underestimated because it is difficult to diagnose. We investigated the significance of *C. burnetii* as the cause of culture-negative endocarditis and vascular infection by examining blood and tissue specimens using serological testing and polymerase chain reaction (PCR).

**Methods:**

All patients with infective endocarditis or large vessel vasculitis were prospectively enrolled at a tertiary-care hospital from May 2016 through September 2020. Q fever endocarditis and vascular infection were diagnosed based on: (1) positive PCR for a cardiac valve or vascular tissue, (2) positive PCR for blood or phase I immunoglobulin G (IgG) ≥ 6400, or (3) phase I IgG ≥ 800 and < 6400 with morphologic abnormality. PCR targeted *C. burnetii* transposase gene insertion element IS*1111a.*

**Results:**

Of the 163 patients, 40 (25%) had culture-negative endocarditis (*n* = 35) or vascular infection (*n* = 5). Of the 40 patients, 24 (60%) were enrolled. Eight (33%) were diagnosed with Q fever endocarditis or vascular infection. Of these 8 patients, 6 had suspected acute Q fever endocarditis or vascular infection with negative phase I IgG. Six patients were not treated for *C. burnetii*, 4 were stable after surgery. One patient died due to surgical site infection after 5 months post-operatively and one died due to worsening underlying disease.

**Conclusions:**

Approximately one-third of patients with culture-negative endocarditis and vascular infection was diagnosed as Q fever. Q fever endocarditis and vascular infection may be underestimated in routine clinical practice in South Korea.

**KEY MESSAGE**
- Q fever endocarditis and vascular infection may be underestimated in routine clinical practice, thus, try to find evidence of *C. burnetti* infection in suspected patients by all available diagnostic tests including PCR.

## Introduction

Culture negative endocarditis is a life-threatening condition associated with significant morbidity and mortality. It accounts for 15–40% of all cases of infective endocarditis [1–3]. There are several causes of culture-negative endocarditis. Of these, infection due to intracellular or non-culturable pathogens remains a diagnostic and therapeutic challenge. *Coxiella burnetii* is the most common causative pathogen [3,4]. Q fever endocarditis is clinically important because the diagnostic delay and the absence of combination treatment can be associated with mortality and serological monitoring is necessary to monitor relapse [5]. In addition, Q fever vascular infection is a disease entity as well-known as Q fever endocarditis, and it is associated with high mortality and major complications [6–9].

The microbiological diagnosis of Q fever endocarditis and vascular infection mainly relies on serology. So, a certain cut-off titre of phase I immunoglobulin G (IgG) antibody with clinically suspected Q fever endocarditis easily makes a diagnosis, although the serology cannot distinguish an acute infection from a past infection [10]. However, the appropriate cut-off value of phase I IgG antibody titre for an accurate diagnosis is contentious. High phase I IgG antibody titre is found in asymptomatic patients with cardiovascular risk, whereas there are patients with documented endocarditis with low titres [11,12]. Serological testing might also be delayed by the time to send samples to a reference laboratory. The development of polymerase chain reaction (PCR) to detect *C. burnetii* DNA in blood, cardiac valves, or other surgical tissue biopsy specimens has helped lessen these problems. Advantages of PCR include early detection, the short turn-around time for results, and high specificity [13]. However, *C. burnetii* DNA may be detected only in the early period of infection [14] with limited sensitivity for the diagnosis of Q fever endocarditis. Despite of this limitation, the positive *C. burnetii* PCR make a diagnosis more definitive. Therefore, there is no single test with a 100% predictive value for Q fever endocarditis or vascular infection. New criteria have recently been proposed incorporating PCR and serological test results [11,12,14].

Little is known about Q fever endocarditis or vascular infection in South Korea [15]. However, the incidence of Q fever has increased from 0.05 to 0.31/100000 population per year in the last 5 years [16]. Presently, we investigated the significance of *C. burnetii* as a causative agent of culture-negative endocarditis and vascular infection in South Korea using serological testing and PCR to detect *C. burnetii* DNA in blood, cardiac valve, and vascular tissue samples.

## Materials and methods

### Study patients

All adult patients with suspected infective endocarditis or vascular infection were prospectively screened between May 2016 and September 2020. The study was conducted in Asan Medical Centre, a 2700-bed, university-affiliated tertiary-care teaching hospital in Seoul, Republic of Korea. Patients with culture-negative endocarditis and vascular infection patients were enrolled in this study. Culture negative infective endocarditis was defined as the absence of microbial growth in blood and cardiac valve tissues culture and meeting definite or possible infective endocarditis according to modified Duke criteria [10]. Culture negative vascular infection was defined as the absence of microbial growth in blood and vascular tissue culture and was proved large vessel or prosthetic infection by imaging techniques that included ^18^F-fluorodeoxyglucose positron emission tomography/computed tomography (^18^F-FDG PET/CT) or computed tomography (CT). Data collected included: demographic variables, information regarding contact with cattle or livestock, predisposing heart disease, history of previous heart surgery procedure, symptoms and signs at presentation, microbiological and imaging findings, surgical intervention, type and duration of antimicrobial therapy, and patient outcome. Informed written consent was obtained from all patients. This study was approved by the Institutional Review Board of Asan Medical Centre (ethical approval number 2016-0748)

### Definition of Q fever endocarditis and vascular infection

Q fever endocarditis and vascular infection were diagnosed with either definitive or possible according to the new recently published criteria [11,14]. Definite criteria included detection of *C. burnetii* by PCR in a cardiac valve, arterial sample or periarterial abscess. Major criteria included morphological abnormalities proven by imaging techniques associated with microbiological evidence. The microbiological evidence was positive PCR of the blood or emboli, or single-phase I IgG antibody titre ≥ 1:6400 by indirect immunofluorescence assay (IFA). Minor criteria included serologic evidence (single-phase I IgG antibody titre ≥ 800 and < 6400 by IFA), non-specific clinical signs of infection, and predisposition to the suspected focus of infection (predisposing heart condition for endocarditis and vascular aneurysm or prosthesis for vascular infection). Definitive diagnosis required fulfilment of; one definite criterion, 2 major criteria, or one major criterion and 3 minor criteria for endocarditis or 2 minor criteria for vascular infection (including one microbiological characteristic and a predisposition). Other cases were considered as possible diagnosis.

### Molecular detection of Coxiella burnetii

#### DNA extraction

To detect *C. burnetii*, DNA was extracted from the blood of patients with suspected Q fever endocarditis or vascular infection. Approximately 4 ml of blood was collected in EDTA tubes and centrifuged at 134 × *g* for 5 min. The plasma was transferred to a sterile tube and kept frozen at −20 °C until further use. Approximately 200 μl of plasma was used for DNA extraction using QIAamp DNA Mini Kit (Qiagen, Hilden, Germany) according to the manufacturer’s instruction with minor modifications. For lysis, AL buffer and proteinase K were added, and samples remained in a water bath for 10 min. Washing with these buffers was done twice and the samples were eluted in 200 μl of AE buffer and stored at −20 °C until use.

DNA was also extracted from a formalin-fixed cardiac valve or arterial tissues. Five sections 5 μm in thickness were cut from each paraffin block and placed in a microtube. Xylene was added, the tube was centrifuged at 13,000 × *g* for 5 min, and the supernatant was discarded. This procedure was repeated 3 times. The specimens were rehydrated through a graded series of ethanol solutions and centrifuged after each washing step. Finally, the tubes were kept open to allow any remaining ethanol to evaporate. DNA was extracted using the Exgene™ FFPE Tissue DNA kit (GeneAll®, Seoul, South Korea) according to the manufacturer’s protocol. Briefly, tissue was digested in FPL buffer and proteinase K in a water bath for 18 h. Samples were washed in BW buffer followed by TW buffer. The DNA was eluted in 100 μl of Tris-Acetate EDTA (TAE) buffer and stored at −20 °C until use.

#### PCR

Detection of *C. burnetii* in blood and tissue by conventional PCR was performed using primers and procedures that were modified from previous reports [17,18]. The gene target was derived from the transposase gene insertion element *IS1111a* of *C. burnetii* RSA 493 (NCBI Nr. NC 002971.4). For each sample, PCR amplification was carried out in two separate assays using different primer sets. Primers (approximately 24 bp) of the first set (1 F; 5′-GAGCGAACCATTGGT ATCG-3′ and 1 R; 5′-CTTTAACAGCGCTTGAACGT-3′’) and the second set (2 F; 5′-CGGGTTAAGCGTGCTCAGTATGTA-3′’ and 2 R; 5′-TGCCACCGCTTTTAATTCCTCCTC-3′’) were synthesised. The conventional PCR process consisted of an initial denaturation step at 95 °C for 15 min; 45 cycles of 95 °C for 30 sec, 57 °C (for first primer set) or 62 °C (for second primer set) for 30 sec, and 72 °C for 30 sec; and a final elongation step at 72 °C for 7 min. Amplification of 5 μl of DNA was performed in a total volume of 25 μl containing 10 × PCR buffer (Qiagen), 2.5 mM MgCl_2_, 0.25 mM deoxynucleotide triphosphate, 25 pmol of each primer, and 1 unit of Taq DNA polymerase (Qiagen). Agarose gel electrophoresis (2%) in the presence of ethidium bromide was used to separate PCR products. The products were visualised using a GelDoc System (Clinx Science Instruments, Shanghai, China). Samples were considered positive if a positive PCR product was observed in two consecutive PCR assays. The presence of *C. burnetii* (*IS1111A* transposase gene, Accession number: MN094854.1) were confirmed by Sanger sequencing according to the study definition in all amplified PCR product.

### Serological methods for Q fever

The blood samples of patients with suspected Q fever endocarditis or vascular infection were sent to the Korea Centres for Disease Control and Prevention (KCDC) for serological testing for *C. burnetii*. The serological testing for *C. burnettii* was performed using a Q Fever IFA IgG kit and IgM kit (Focus Diagnostics, Inc., Cypress, CA, USA), as per the manufacturer’s instructions. In brief, 1:16 screening dilutions for IgG antibody assay were prepared by mixing 1 part patient serum with 15 parts 1 × IgG Sample Diluent Working Solution. Other 1:16 screening dilutions for IgM antibody assay were prepared by mixing 5 μl of patient serum with 75 μl IgM Pre-treatment Diluent. To determine endpoint titres, reconstituted phosphate-buffered saline (PBS) was used to serially dilute the screening dilution and Positive control (supplied in the kit). Q fever substrate slides were removed from cold storage and allowed to reach room temperature. Approximately 25 μl of each serial dilution of the screening dilutions and Positive control were individually placed on an appropriate slide well. The same amount of Negative control (supplied in the kit) was also applied to an appropriate slide well. The slides were incubated in a humid chamber for 30 min (for IgG antibody assay) or 90 min (for IgM antibody assay) at 37 °C. The slides were washed three times for 5 min with PBS using roll-mixer, followed by distilled water, and allowed to air dry. Approximately 25 μl of fluorescein labelled goat anti-human gamma-chain specific IgG or mu-chain specific IgM (supplied in the kit) were applied to each slide well. The slides were incubated in a humid chamber for 30 min at 37 °C and washed as mentioned above. The slides were observed using a fluorescence microscope (ZEISS AXioskop2, Carl Zeiss, Munich, Germany) at a magnification of × 400.

### Measurement of anti-cardiolipin IgG antibodies by enzyme-linked immunosorbent assay (ELISA)

According to the manufacturer’s protocol, the presence of anti-cardiolipin IgG in stored plasma of 8 patients with Q fever endocarditis or vascular infection was measured with a commercial ELISA kit (Novus Biologicals, Centennial, CO, USA). The optical density value of 450 nm (OD450) was measured. When OD450 (sample/negative control) was 2.1 or higher, it was considered as positive for anti-cardiolipin IgG.

### Statistical analyses

Qualitative data were expressed as absolute and relative frequencies. Quantitative data were expressed as medians and interquartile ranges. Fisher’s exact test was used to test the difference between proportions for categorical variables and Mann-Whitney *U*-test for continuous variables. A *p*-value ≤ .05 was considered statistically significant. Statistical analysis was conducted using SPSS software v24.0 (IBM, Armonk, NY, USA).

## Results

**Figure 1.**
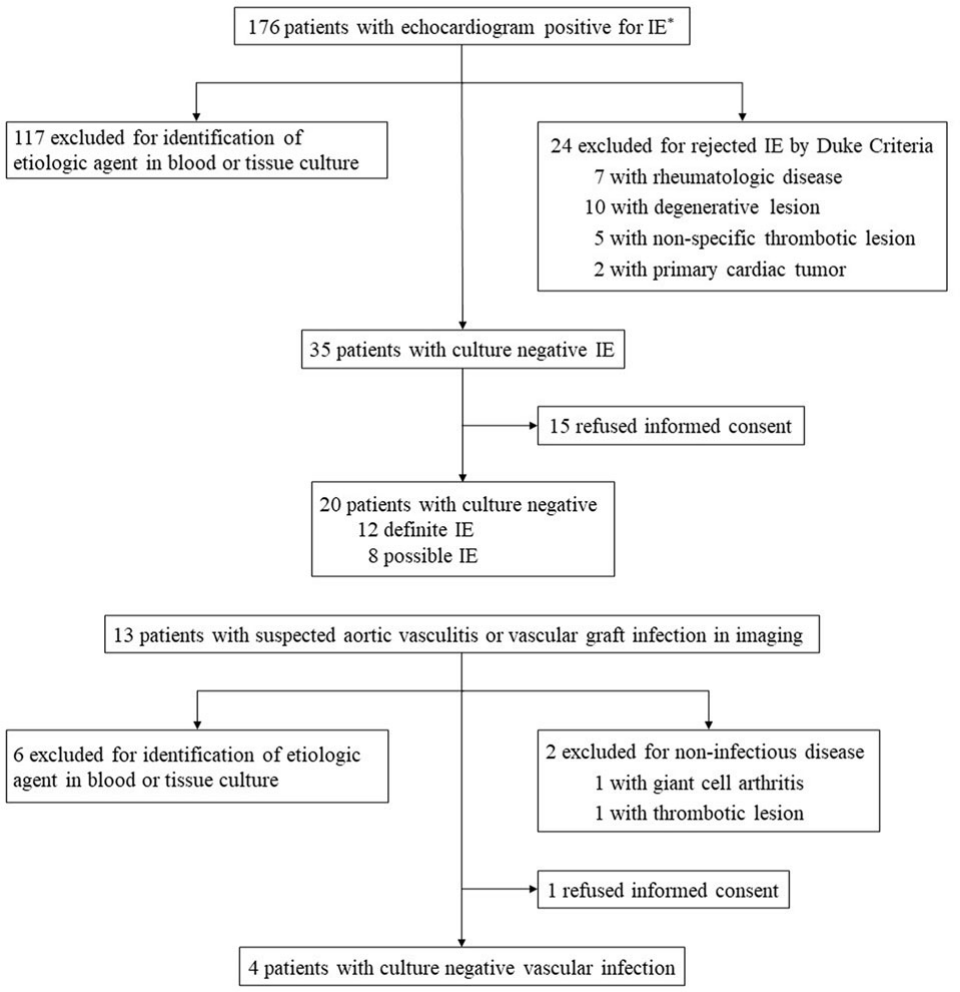
Distribution of the 189 patients with echocardiogram positive for infective endocarditis (IE) or vascular inflammatory lesion *Echocardiogram positive for IE was defined as follows: oscillating intracardiac mass on the valve or supporting structures, in the path of regurgitant jets, or on implanted material in the absence of an alternative anatomic explanation; abscess; or new partial dehiscence of prosthetic valve or new valvular regurgitation.

A total of 176 patients with suspected endocarditis were detected by transthoracic echocardiography (TTE) or transesophageal echocardiography (TEE) and 13 patients with suspected vascular infection were detected by clinical imaging. Twenty-four (14%) were eventually classified as rejected infective endocarditis according to the modified Duke criteria and 2 (15%) excluded due to an alternative diagnosis. Of the remaining total of 163 patients, 123 were excluded due to an infectious agent identified on blood culture or tissue culture. Of the remaining 40 (25%), the diagnosis was culture-negative endocarditis in 35 patients and vascular infection in 5. Twenty-four of the 40 (60%) patients who provided informed consent were finally enrolled (Figure 1).

Tables 1 and 2 summarise the clinical characteristics, echocardiographic findings, clinical imaging findings, histologic features of excised tissue, and the results of serology and PCR to detect *C. burnetii* from blood or tissue in the 24 patients with culture-negative endocarditis and vascular infection. The median age [interquartile range] was 61 [44–72] years, 19 (79%) were male, and 11 (46%) had underlying predisposing conditions. Based on the modified Duke criteria, 12 patients were classified as definitive infective endocarditis and 8 patients as possible infective endocarditis. The use of antimicrobial agents prior to blood culture was recorded in 6 patients (25%). The median period of use was 9 [4–20] days. The median follow-up time at the date of the last disease assessment was 207 [89–476] days. A total of 13 (54%) received surgical treatment due to valvular insufficiency, valve perforation, or other complication. All but 2 patients received empirical antibacterial therapy with a median duration of use of 43 [26–66] days. Four (17%) patients died during the follow-up period.

**Table 1.** Clinical characteristics of 8 patients with Q fever endocarditis and vascular infection and results of Q fever serologic testing and PCR for *Coxiella burnetii* from blood or tissue.

No.	Sex/ Age	Duke classification	Comorbid conditions	Predisposing heart condition	Symptoms	Infected structure	Image findings	Histologic features	Acute serology^†^	Convalescent serology^‡^	PCR from blood	PCR from tissue
1	M/20	Definite IE	None	None	Fever, hepatitis, acute embolic cerebral infarction, endophthalmitis	MV	Vegetation, cardiac abscess (TTE/TEE)	Acute and chronic valvulitis with abscess formation and necrosis	<16/<16/ NA/NA	NA	Negative	Positive
2	M/73	Definite IE	HTN	AVR d/t bicuspid AV	Fever, multiple mycotic aneurysm, vertebral osteomyelitis	AV	Vegetation, valvular thickening, cardiac abscess (TTE/TEE)	Subacute necrotising valvulitis with vegetation	<16/<16/ <16/<16	NA	Negative	Positive
3	F/82	Definite IE	None	Bicuspid AV	Fever, renal involvement	AV	Vegetation, valve perforation, valvular insufficiency (TTE/TEE)	Necrotising inflammation with calcification	<16/16/ <16/<16	NA	Positive	Negative
4	M/40	Definite IE	None	None	Fever, acute embolic cerebral infarction, splenic/renal infarction, splenomegaly	MV	Vegetation, valvular insufficiency (TTE/TEE)	NA	<16/4096/ 512/8192	<16/4096/ 2048/8192	Negative	NA
5	M/61	Definite IE	None	AVR and MV repair d/t severe rheumatic valvular disease	Dyspnoea	AV	New partial dehiscence of prosthetic AV, valvular insufficiency, cardiac abscess (TTE/TEE)	Subacute valvulitis, mid with surface fibrinous exudate	<16/<16/ <16/<16	<16/<16/ <16/<16	Negative	Positive
6	M/44	Possible IE	Malignancy	None	Fever, acute embolic cerebral infarction, renal infarction	MV	Vegetation, valvular insufficiency (TTE/TEE)	NA	<16/<16/ <16/<16	NA	Positive	NA
7	F/47	NA	None	TEVAR d/t aortic dissection	Fever, left pleuritic chest pain	Aorta	Complicated haematoma (mycotic aneurysm) of the ruptured false lumen wall of the descending thoracic aorta (CT)	Aortic dissection with thrombus and fibrinous materials	<16/16/ <16/<16	<16/<16/ <16/<16	Negative	Positive
8	M/68	NA	None	None	Fever, hepatitis, splenomegaly	Aorta	Newly appeared mild hypermetabolic activity in ascending aortal wall (PET-CT)	NA	512/64/ 64/<16	>2048/>2048/ 256/2048	Positive	NA

^†^Serology results (phase II IgM/phase II IgG/phase I IgM/phase I IgG titre) of blood samples drawn at the time of initial screening (acute phase). ^‡^Serology results (phase II IgM/phase II IgG/phase I IgM/phase I IgG titre) of blood samples drawn 3–6 weeks apart from the initial blood sampling (convalescent phase). Abbreviations: AS, aortic valve stenosis; AV, aortic valve; AVR, aortic valve replacement; COPD, chronic obstructive pulmonary disease; CT, computed tomography; DM, diabetes mellitus; F, female; HTN, hypertension; IE, infective endocarditis; M, male; MV, mitral valve; MVR, mitral valve replacement; NA, not available; PET-CT, positron emission tomography-computed tomography; PV, pulmonic valve; TAVR, transcatheter aortic valve replacement; TEE, transesophageal echocardiography; TEVAR, thoracic endovascular aortic repair; TTE, transthoracic echocardiography; VSD, ventricular septal defect.

**Table 2.** Clinical characteristics of 16 patients with culture-negative infective endocarditis (IE) or vascular infection without the microbiologic evidence for Q fever.

No.	Sex/ Age	Duke classification	Comorbid conditions	Predisposing heart condition	Symptoms	Infected structure	Image findings	Histologic features	Acute serology^†^	Convalescent serology^‡^	PCR from blood	PCR from tissue
1	M/33	Definite IE	None	VSD	Fever, splenomegaly	PV, AV	Vegetation, valvular thickening, valvular insufficiency (TTE/TEE)	Chronic and acute necrotising inflammation	<16/<16/ NA/NA	NA	Negative	Negative
2	F/63	Definite IE	None	None	Fever, acute embolic cerebral infarction,	MV	Vegetation, valvular insufficiency (TTE/TEE)	Necrotising inflammation	<16/16/ <16/<16	NA	Negative	Negative
3	M/50	Definite IE	None	Bentall operation and MVR d/t Severe rheumatic valvular disease	Fever, acute embolic cerebral infarction, endopththalmitis, brain abscess	MV, AV	Vegetation, valvular thickening (TTE/TEE)	NA	<16/16/<16/ <16	<16/16/ <16/<16	negative	NA
4	M/71	Definite IE	None	AVR d/t Severe degenerative AS with bicuspid AV	Febrile sense	AV	Valvular thickening, New valvular insufficiency (TTE/TEE)	Suture granuloma with necrotic tissue	<16/16/ <16/<16	<16/16/ <16/<16	Negative	Negative
5	M/22	Definite IE	None	None	Fever, acute embolic cerebral infarction, splenic infarction, splenomegaly	MV	Vegetation, valvular insufficiency (TTE/TEE)	Necrotising inflammation with reactive fibroblastic and histiocytic infiltration	<16/16/ <16/<16	NA	Negative	Negative
6	M/56	Definite IE	COPD	Bicuspid AV	Fever	AV	Vegetation, valvular insufficiency (TTE/TEE)	Many yeast-form fungal organisms with fibrin at valvular surface	<16/16/ <16/<16	<16/16/ <16/<16	Negative	Negative
7	F/80	Definite IE	DM	None	Dyspnoea	AV	Valvular thickening, New valvular insufficiency (TTE/TEE)	Acute necrotising valvulitis	<16/16/ <16/<16	<16/16/ <16/<16	Negative	Negative
8	M/82	Possible IE	None	None	Fever, hepatitis, acute embolic cerebral infarction, endophthalmitis	MV	Vegetation, valvular thickening (TTE/TEE)	NA	<16/<16/ NA/NA	NA	Negative	NA
9	M/60	Possible IE	Malignancy	None	Fever,	MV, AV	Vegetation, valvular thickening, valvular insufficiency (TTE/TEE)	NA	<16/16/ <16/<16	NA	Negative	NA
10	M/36	Possible IE	None	None	Fever, acute embolic cerebral infarction, splenic/renal infarction, mycotic aneurysm, splenomegaly	MV	Vegetation, valve perforation, mitral valve cordae tendinae rupture, valvular insufficiency (TTE/TEE)	NA	<16/16/ <16/<16	NA	Negative	NA
11	M/70	Possible IE	DM, COPD	None	Dyspnoea, septic pulmonary infarct	MV	Vegetation, valvular thickening, Mitral valve cordae tendinae rupture	NA	<16/16/ <16/<16	<16/16/ <16/<16	Negative	NA
12	M/63	Possible IE	Malignancy, DM	None	Fever, dyspnoea, acute embolic cerebral infarction, hepatitis	MV	Vegetation, valvular insufficiency (TTE/TEE)	NA	<16/16/ <16/<16	NA	Negative	NA
13	M/83	Possible IE	DM, HTN	None	Fever, acute embolic cerebral infarction, mycotic aneurysm	MV	Vegetation, calcification (TTE/TEE)	NA	<16/16/ <16/<16	NA	Negative	NA
14	F/88	Possible IE	HTN	TAVR d/t Severe degenerative AS	Fever	AV	Vegetation, valvular thickening (TTE/TEE)	NA	<16/16/ <16/<16	<16/16/ <16/<16	Negative	NA
15	M/43	NA	None	Total arch replacement d/t aortic dissection and congenital aorta anomaly	Fever, splenomegaly	Aorta	Active inflammatory focus suggesting graft infection at mid aortic arch (PET-CT)	NA	<16/16/ <16/<16	NA	Negative	NA
16	M/50	NA	DM	Total arch replacement d/t aortic dissection	Fever, epigastric pain	Aorta	Partially thrombosed saccular pseudoaneurysm (mycotic aneurysm) of descending thoracic aorta due to rupture of false lumen with suspicious communication of oesophagus and pseudoaneurysm (CT)	Fistula between oesophagus and pseudoaneurysm of aorta with abscess, subserositis with subserosal fibrosis	<16/16/ <16/<16	<16/16/ <16/<16	Negative	Negative

^†^Serology results (phase II IgM/phase II IgG/phase I IgM/phase I IgG titre) of blood samples drawn at the time of initial screening (acute phase). ^‡^Serology results (phase II IgM/phase II IgG/phase I IgM/phase I IgG titre) of blood samples drawn 3–6 weeks apart from the initial blood sampling (convalescent phase). Abbreviations: AS, aortic valve stenosis; AV, aortic valve; AVR, aortic valve replacement; COPD, chronic obstructive pulmonary disease; CT, computed tomography; DM, diabetes mellitus; F, female; HTN, hypertension; IE, infective endocarditis; M, male; MV, mitral valve; MVR, mitral valve replacement; NA, not available; PET-CT, positron emission tomography-computed tomography; PV, pulmonic valve; TAVR, transcatheter aortic valve replacement; TEE, transesophageal echocardiography; TEVAR, thoracic endovascular aortic repair; TTE, transthoracic echocardiography; VSD, ventricular septal defect.

Of the 24 patients with culture-negative infective endocarditis or vascular infection, 8 (33%) were diagnosed as Q fever endocarditis or vascular infection (Table 1). All patients except one did not have zoonotic risk factors. There were no significant differences regarding comorbidities, predisposing heart conditions, the proportion of patients requiring surgical intervention, duration of antimicrobial agent therapy, and all-cause of mortality between patients diagnosed with Q fever infection and the rest of the patients (Table S1). All but two of the patients (Table 1, #4 and #8) did not receive a combination of doxycycline and hydroxychloroquine. Two patients (Table 1, #1 and #7) recovered after undergoing cardiac valve repair surgery and descending aortic graft replacement surgery, respectively. Two patients (Table 1, #3 and #5) remained stable with moderate paravalvular regurgitation. Two patients died. One (Table 1, #2) died due to the development of surgical site infection 5 months post-operatively. The other (Table 1, #6) died due to worsening underlying disease.

Eight patients with Q fever endocarditis or vascular infection were classified as definite diagnosis including 4 patients with positive infected tissue PCR, 2 patients with positive blood PCR, one patient with both positive blood PCR and phase I IgG titre ≥ 800, and one patient with phase I IgG titre ≥ 6400. PCR from blood samples was performed for all enrolled patients, and PCR from tissue samples was performed for 12 of 13 patients who received surgical treatment. Blood samples for PCR were collected at a median of 28 [10–51] days from the onset of symptoms and 10 [6–18] days from the detection of abnormality by clinical imaging. Tissue samples for PCR were collected at a median of 39 [23–94] days from the onset of symptoms and 12 [5–28] days from the detection of abnormality by clinical imaging. IFA serological testing was performed for all enrolled patients. Blood samples for serological testing were collected at a median of 24 [10–43] days from the onset of symptoms and 7 [4–16] days from the detection of abnormality by clinical imaging. Phase I or II antibodies were not detected in 22 of the 24 patients at initial blood sampling. Eleven patients underwent Q fever serological testing during the covalence phase. Blood samples of the convalescent phase were collected at a median of 25 [22‒34] days from the collection of first blood samples. Of 11 patients, 7 revealed negative Q fever PCR results (#3, #4, #6, #7, #11, #14, #16 in Table 2). The rest of the 4 patients were finally diagnosed Q fever endocarditis or vascular infection, including 2 patients with phase I IgG titre ≥ 800 (#4, #8 in Table 1) and 2 patients without Q fever serological response (#5, #7 in Table 1). One patient displayed seroconversion in phase I IgG (Table 1, #8). Taken together, there were 6 and 2 patients who had molecular evidence of Q fever endocarditis or vasculitis without the evidence of *C. burnetii* antibody response during the acute phase and convalescent period, respectively (Table 1). The presence of anti-cardiolipin IgG supporting the diagnosis of acute Q fever endocarditis was determined in 8 patients with Q fever endocarditis or vascular infection. One of the 8 patients (Table 1, #8) were positive for anti-cardiolipin IgG.

One patient (Table 1, #8) fulfilled the microbiologic criteria for Q fever (positive blood PCR and phase I IgG titre ≥ 800). The patient was ultimately judged to have Q fever vascular infection because ^18^F-FDG PET/CT scan revealed focal hypermetabolic activity in ascending aorta, even though he did not have a vascular prosthesis and did not display aneurysmal change of the involved area. He was a 68-year old farmer with no underlying diseases. He was admitted because of a 3-week history of fever and was diagnosed with acute Q fever based on serologic results for *C. burnetii* (phase II IgM/IgG and phase I IgM/IgG antibodies titre of 512/64 and 64/<16, respectively). Baseline ^18^F-FDG PET/CT performed on the admission date demonstrated no abnormal lesions. After a 14-day administration of doxycycline, the fever persisted and the phase I IgG antibody titre had risen to 512. TEE was performed on suspicion of Q fever endocarditis. No abnormality was apparent. Hydroxychloroquine was added in case of evolution to persistent Q fever infection. Despite the combination treatment for 7 days, the fever persisted and the phase I IgG antibody titre rose to 2048. Another ^18^F-FDG PET/CT revealed newly appearing focal hypermetabolic activity in the ascending aorta, suggestive of the presence of a large vessel vasculitis. He recovered while being maintained on the combination treatment without surgery and was loss to follow-up after discharge.

## Discussion

The present study investigated Q fever endocarditis and vascular infection cases in culture negative infective endocarditis and vascular infection in South Korea. Among 163 patients with infective endocarditis or vascular infection, 40 had negative results in blood and tissue culture. Of the 40, 24 of them were included in the analysis. Finally, 8 (33%) patients with culture negative endocarditis and vascular infection were diagnosed as Q fever by serological and molecular testing.

The incidence of *C. burnetii* infection in culture-negative endocarditis varies widely, ranging from 2.5% to 48%, depending on detecting methods and study design [2–4]. In this study, 6 patients diagnosed with Q fever endocarditis and vascular infection fulfilled the microbiologic criteria based on PCR results but did not show a concomitant increase in phase I IgG antibody titre. There could be concerns about false-positive results regarding the diagnostic value of the PCR-based method. The specificity of PCR detecting *C. burnetii* DNA in persistent Q fever infection has been rarely reported. In one study from France, 100 sera from 100 patients with endocarditis caused by other microorganisms were tested to estimate the specificity of their in-house PCR targeting *IS1111* of *C. burneti*i. The authors reported that all PCR results were negative [13]. We also performed in-house PCR targeting *IS1111*a of *C. burnetii* in valve tissues from 20 control patients with culture-positive endocarditis to check for potential false-positive results in a previous study [18]. Of the 20 control patients, none showed a positive Q fever PCR results from cardiac valve tissue. Therefore, the possibility of false-positive PCR results is low. On the other hand, there could be concerns about the diagnostic accuracy of serological testing. Since the blood samples were submitted to the national reference laboratory during the entire study period and were performed by the serologic test using the commercial kit according to the manufacturer’s instructions, the possibility of inaccurate serologic test results is low. Therefore, we cautiously assumed that these patients might have “acute Q fever endocarditis and vascular infection.”

The repeated tests for phase I IgG antibody are highly sensitive for the diagnosis Q fever endocarditis except in immunocompromised hosts, those with massive transfusion, or those with acute Q fever endocarditis. So, Q fever infection could not be excluded without repeated tests especially in those with suspected acute Q fever endocarditis, even with a phase I IgG antibody titre < 800, given the reports of several such cases [5,6,19]. In addition, acute Q fever endocarditis is an emerging clinical entity as it was suggested that primary infection caused by *C. burnetii* could lead to cardiac valve infection [20,21]. Of 8 patients who were classified as Q fever endocarditis or vascular infection, (1) 4 did not undergo blood sampling in the convalescent phase, (2) 2 showed no serological response even from the convalescent blood samples, and (3) 2 demonstrated phase I IgG titre ≥ 800 in the acute or convalescent phase. Unfortunately, it could not be directly verified whether the serological response developed later or not because of the absence of the convalescent blood samples from these 4 patients. We remained the assumption that there may be insufficient time for the serological response to occur in these 4 patients.

On the other hand, there were 2 patients without Q fever serologic response even in the blood samples collected at the convalescent phase. Furthermore, negative phase II serology in acute Q fever endocarditis is unusual, although phase I serology may be negative or low titre [21]. It has been established that genetic differences between *C. burnetti* strains could affect their virulence and host adaptation [22,23]. Results from studies in the guinea pigs infection model implied that *C. burnetii* strains with different genetic profiles could show a variable range of the magnitude of *C. burnetti*-specific IgG level detected by the commercial IFA kit using antigens of Nine Mile strain [23]. Therefore, we carefully could be assumed that certain *C. burnetti* strains to evoke the serologic response not detected by commercial kit using Nine Mile antigen could exist. As far as we know, only a few studies have been reported about the genetic profiles of *C. burnetii* strains in South Korea [24]. Additional studies are needed to further investigate the genetic diversity and pathogenicity of *C. burnettii* circulating in South Korea.

Recently, it was suggested that a single-phase I IgG cut-off definition for Q fever infection is not possible and that new diagnostic criteria based on molecular or serological assay are necessary [11,12,14]. The present results support this suggestion. Of note, increased anti-cardiolipin antibodies can be helpful in identifying acute Q fever patients who eventually progress to acute endocarditis [21]. One of the 8 patients with Q fever endocarditis or vascular infection was positive for anti-cardiolipin IgG in this study. Although it was lower values with respect to those reported by the French group (anti-cardiolipin IgG levels were elevated in all 9 patients with acute Q fever endocarditis in their previous study, and positive anti-cardiolipin antibody was shown 68% [28/41] of acute Q fever endocarditis in their later study) [20,21], it could be helpful in identifying a patient who eventually progressed to acute Q fever vasculitis (Table 1, #8). Further studies are needed on appropriate diagnostic tests to detect acute Q fever endocarditis.

One patient was diagnosed with Q fever vascular infection based on the findings in ^18^F-FDG PET/CT scan even though he did not have a vascular prosthesis and did not display aneurysmal change of the involved area. Q fever vascular infection can develop after primary *C. burnetti* infection in patients with predisposing factors that include prosthesis or aneurysm, based mainly on case reports [6]. However, several cases of Q fever infection associated vasculitis without aneurysmal change have been described [25–28]. ^18^F-FDG PET/CT is useful to screen for early detection of Q fever vascular infection in the risk group [29] and is a promising diagnostic tool for localisation of persistent focalised Q fever infection [30]. In addition, anti-cardiolipin antibiotics associated with acute Q fever endocarditis were positive. Therefore, we suggest that this patient could have had acute Q fever vasculitis or was in the early stage of persistent Q fever vascular infection.

This study has several limitations. First, it was possible that the small number of more severe cases were only included since this was a single centre study at a major referral hospital. Second, since the modified Duke criteria were used to define culture-negative endocarditis, most of the morphological abnormalities detected by echocardiography were valvular vegetation. However, since valvular vegetation was reported to occur in only approximately 30% of patients with Q fever endocarditis [5], the incidence of Q fever endocarditis could be underreported in this study. Third, we did not perform the serological or molecular testing for other common microbial aetiologies of culture-negative endocarditis, such as *Bartonella, Legionella, Mycoplasma* or *Chlamydia* species. Concerning the molecular cross-reactivity between *Bartonella* species and *C. burnetii* [2–4], further study is necessary to include this missed diagnosis, since *Bartonella* endocarditis also had been reported in South Korea [31]. Fourth, we could not perform the additional genetic analysis for confirmed Q fever cases because of a small number of patients and low amount of genomic DNA. Further study with large cohort samples would be desired for the investigation of the genetic epidemiology of *C. burnetii* in South Korea using molecular analysis such as multispare sequence typing. Fifth, the follow-up period was relatively short in the evaluation of the prognosis of patients with Q fever endocarditis and vascular infection. Finally, there was insufficient information to explain the serological response since the convalescent blood samples were obtained only from some patients.

In summary, approximately one-third of patients with culture-negative endocarditis and vascular infection was diagnosed as Q fever using a Q fever serological test and PCR. The finding suggests that Q fever endocarditis and vascular infection has been underestimated in routine clinical practice in South Korea. Given this high prevalence, physicians should suspect *C. burnetii* as a causative agent of culture-negative endocarditis and vascular infection, even if the patient does not have zoonotic risk factors. In addition, regarding the difficulties of its diagnosis, physicians should try to find evidence of *C. burnetti* infection by all available diagnostic tests when the infection is suspected.

## Supplementary Material

Supplemental Material

## Data Availability

The authors confirm that the data supporting the findings of this study are available within the article and its supplementary materials.

## References

[1] Díez-Villanueva P, Muñoz P, Marín M, et al. Infective endocarditis: absence of microbiological diagnosis is an independent predictor of inhospital mortality. Int J Cardiol. 2016;220:162–165. 27379918 10.1016/j.ijcard.2016.06.129

[2] Lamas CC, Fournier PE, Zappa M, et al. Diagnosis of blood culture-negative endocarditis and clinical comparison between blood culture-negative and blood culture-positive cases. Infection. 2016;44(4):459–466. 26670038 10.1007/s15010-015-0863-x

[3] Fournier PE, Gouriet F, Casalta JP, et al. Blood culture-negative endocarditis: improving the diagnostic yield using new diagnostic tools. Medicine. 2017;96(47):e8392. 29381916 10.1097/MD.0000000000008392 PMC5708915

[4] Fournier PE, Thuny F, Richet H, et al. Comprehensive diagnostic strategy for blood culture-negative endocarditis: a prospective study of 819 new cases. Clin Infect Dis. 2010;51(2):131–140. 20540619 10.1086/653675

[5] Million M, Thuny F, Richet H, et al. Long-term outcome of Q fever endocarditis: a 26-year personal survey. Lancet Infect Dis. 2010;10(8):527–535. 20637694 10.1016/S1473-3099(10)70135-3

[6] Wegdam-Blans MC, Vainas T, van Sambeek MR, et al. Vascular complications of Q-fever infections. Eur J Vasc Endovasc Surg. 2011;42(3):384–392. 21622013 10.1016/j.ejvs.2011.04.013

[7] Karhof S, van Roeden SE, Oosterheert JJ, et al. Primary and secondary arterial fistulas during chronic Q fever. J Vasc Surg. 2018;68(6):1906–1913.e1. 29685511 10.1016/j.jvs.2018.01.044

[8] Buijs SB, Stuart SK, Oosterheert JJ, et al. Long-term serological follow-up after primary *Coxiella burnetii* infection in patients with vascular risk factors for chronic Q fever. Eur J Clin Microbiol Infect Dis. 2021;40(7):1569–1572. 33566203 10.1007/s10096-021-04179-5 PMC8205920

[9] Puges M, Bérard X, Caradu C, et al. Polymicrobial infections among patients with vascular Q fever, France, 2004-2020. Emerg Infect Dis. 2021;27(7):1961–1963. 34152966 10.3201/eid2707.210282 PMC8237867

[10] Li JS, Sexton DJ, Mick N, et al. Proposed modifications to the duke criteria for the diagnosis of infective endocarditis. Clin Infect Dis. 2000;30(4):633–638. 10770721 10.1086/313753

[11] Raoult D. Chronic Q fever: expert opinion versus literature analysis and consensus. J Infect. 2012;65(2):102–108. 22537659 10.1016/j.jinf.2012.04.006

[12] Wegdam-Blans MC, Kampschreur LM, Delsing CE, et al. Chronic Q fever: review of the literature and a proposal of new diagnostic criteria. J Infect. 2012;64(3):247–259. 22226692 10.1016/j.jinf.2011.12.014

[13] Fenollar F, Fournier PE, Raoult D. Molecular detection of *Coxiella burnetii* in the sera of patients with Q fever endocarditis or vascular infection. J Clin Microbiol. 2004;42(11):4919–4924. 15528674 10.1128/JCM.42.11.4919-4924.2004 PMC525147

[14] Eldin C, Mélenotte C, Mediannikov O, et al. From Q fever to *Coxiella burnetii* infection: a paradigm change. Clin Microbiol Rev. 2017;30(1):115–190. 27856520 10.1128/CMR.00045-16 PMC5217791

[15] Moon S-Y, Choi YS, Park M-Y, et al. Two cases of Q fever endocarditis. Infect Chemother. 2009;41(3):199–204.

[16] Korean Disease Control and Prevention Agency (KDCA), Infectious Disease Portal. [cited 2020 October 24]. Available from: http://www.kdca.go.kr/npt/biz/npp/ist/simple/simplePdStatsMain.do

[17] Bae M, Jin CE, Park JH, et al. Diagnostic usefulness of molecular detection of *Coxiella burnetii* from blood of patients with suspected acute Q fever. Medicine (Baltimore)). 2019;98(23):e15724. 31169672 10.1097/MD.0000000000015724 PMC6571429

[18] Jang YR, Song JS, Jin CE, et al. Molecular detection of *Coxiella burnetii* in heart valve tissue from patients with culture-negative infective endocarditis. Medicine. 2018;97(34):e11881. 30142785 10.1097/MD.0000000000011881 PMC6112960

[19] Grisoli D, Million M, Edouard S, et al. Latent Q fever endocarditis in patients undergoing routine valve surgery. J Heart Valve Dis. 2014;23(6):735–743. 25790621

[20] Melenotte C, Epelboin L, Million M, et al. Acute Q fever endocarditis: a paradigm shift following the systematic use of transthoracic echocardiography during acute Q fever. Clin Infect Dis. 2019;69(11):1987–1995. 30785186 10.1093/cid/ciz120

[21] Million M, Thuny F, Bardin N, et al. Antiphospholipid antibody syndrome with valvular vegetations in acute Q fever. Clin Infect Dis. 2016;62(5):537–544. 26585519 10.1093/cid/civ956

[22] Russell-Lodrigue KE, Andoh M, Poels MW, et al. *Coxiella burnetii* isolates cause genogroup-specific virulence in mouse and guinea pig models of acute Q fever. Infect Immun. 2009;77(12):5640–5650. 19786560 10.1128/IAI.00851-09 PMC2786457

[23] Long CM, Beare PA, Cockrell DC, et al. Comparative virulence of diverse *Coxiella burnetii* strains. Virulence. 2019;10(1):133–150. 30782062 10.1080/21505594.2019.1575715 PMC6389282

[24] Kim YH, Kim D. Genotyping of *Coxiella burnetii* strains detected in cattle from a nationwide survey in Korea. J Vet Sci. 2019;20(1):95–97. 30541186 10.4142/jvs.2019.20.1.95 PMC6351771

[25] Baziaka F, Karaiskos I, Galani L, et al. Large vessel vasculitis in a patient with acute Q-fever: a case report. IDCases. 2014;1(3):56–59. 26952153 10.1016/j.idcr.2014.07.004 PMC4762790

[26] de Worm S, Giot JB, Courtoy C, et al. A case of giant cell arteritis associated with culture-proven *Coxiella burnetii* aortitis. Int J Infect Dis. 2018;69:50–54. 29408476 10.1016/j.ijid.2018.01.028

[27] Lefebvre M, Grossi O, Agard C, et al. Systemic immune presentations of *Coxiella burnetii* infection (Q fever). Semin Arthritis Rheum. 2010;39(5):405–409. 19110298 10.1016/j.semarthrit.2008.10.004

[28] Odeh M, Oliven A. Temporal arteritis associated with acute Q fever. A case report. Angiology. 1994;45(12):1053–1057. 7985833 10.1177/000331979404501209

[29] Wegdam-Blans MC, Stokmans RA, Tjhie JH, et al. Targeted screening as a tool for the early detection of chronic Q fever patients after a large outbreak. Eur J Clin Microbiol Infect Dis. 2013;32(3):353–359. 23010905 10.1007/s10096-012-1749-9

[30] Eldin C, Melenotte C, Million M, et al. 18F-FDG PET/CT as a central tool in the shift from chronic Q fever to *Coxiella burnetii* persistent focalized infection: a consecutive case series. Medicine. 2016;95(34):e4287. 27559944 10.1097/MD.0000000000004287 PMC5400310

[31] Lim MH, Chung DR, Kim WS, et al. First case of *Bartonella quintana* endocarditis in Korea. J Korean Med Sci. 2012;27(11):1433–1435. 23166430 10.3346/jkms.2012.27.11.1433 PMC3492683

