# Supplementary material for: Molecular diagnosis of Coxiella burnetii in culture negative endocarditis and vascular infection in South Korea
Source: Ann Med. 2021 Nov 22;53(1):2258–67. doi: 10.1080/07853890.2021.2005821 (PMC8805875; doi:10.1080/07853890.2021.2005821)
Supplement: Supplemental Material [file IANN_A_2005821_SM1401.docx]

**Table S1. Clinical and demographic characteristics of 24 patients with culture negative infective endocarditis or vascular infection.**

|  | **All patients**  **(n=24)** | **Patients with Q fever**  **endocarditis and**  **vascular infection**  **(n=8)** | **Patients without**  **microbiologic evidence**  **for Q fever**  **(n=16)** | ***p***  **value** |
| --- | --- | --- | --- | --- |
| Age^‡^, years | 61 [44‒72] |  |  | 0.57 |
| Male^§^ | 19 (79) | 6 (75) | 13 (81) | 0.99 |
| Comorbidity^§^ | 10 (42) | 2 (25) | 8 (50) | 0.39 |
| Predisposing heart condition^§^ | 11 (46) | 4 (50) | 7 (44) | 0.99 |
| Received antimicrobial  therapy before blood culture^§^ | 6 (25) | 3 (38) | 3 (19) | 0.36 |
| Infected structure^§†^ |  |  |  | 0.81 |
| Mitral valve | 12 (44) | 3 (38) | 9 (47) | 0.70 |
| Aortic valve | 10 (37) | 3 (38) | 7 (37) | 0.99 |
| Pulmonary valve | 1 (4) | 0 (0) | 1 (5) | 0.99 |
| Tricuspid valve | 0 (0) | 0 (0) | 0 (0) |  |
| Other structure | 4 (15) | 2 (24) | 2 (11) | 0.56 |
| Valvular dysfunction^§^ | 13 (54) | 4 (50) | 9 (56) | 0.99 |
| Received surgical treatment^§^ | 13 (54) | 5 (63) | 8 (50) | 0.68 |
| Duration of antimicrobial  therapy^‡^, days | 43 [26‒66] | 61 [44‒89] | 64 [13‒57] | 0.05 |
| Follow-up period^‡^, days | 207 [89‒476] | 167 [75‒246] | 301 [124‒510] | 0.29 |
| All-cause of mortality^§^ | 4 (17) | 2 (25) | 2 (13) | 0.58 |

^†^Multiple valve involvement confirmed in 3 patients.

^‡^Quantitative variables were expressed with median [IQR] and statistic with Mann-Whitney U-test. ^§^Categorical variable was expressed with number (percentage) and statistic with Fisher's exact test.
